# Efficacy and Safety of Eplerenone for Treating Chronic Kidney Disease: A Meta-Analysis

**DOI:** 10.1155/2023/6683987

**Published:** 2023-03-09

**Authors:** Honglei Hu, Mengdie Cao, Yao Sun, Xingqian Jin, Xiaodong Zhao, Xiangguo Cong

**Affiliations:** ^1^Department of Endocrinology, Shandong Zibo Central Hospital, Zibo 255000, China; ^2^Department of Endocrinology, The Affiliated Suzhou Hospital of Nanjing Medical University, Suzhou Municipal Hospital, Suzhou 215000, China

## Abstract

**Background:**

In recent years, a large amount of clinical evidence and animal experiments have demonstrated the unique advantages of mineralocorticoid receptor antagonists (MRA) for treating chronic kidney disease (CKD).

**Aims:**

Accordingly, the present study aimed to systematically assess the second-generation selective MRAs eplerenone's safety and effectiveness for treating CKD.

**Methods:**

Four databases (PubMed, The Cochrane Library, Embase, and Web of Science) were searched for randomized controlled trials (RCT) correlated with eplerenone for treating CKD up to September 21, 2022. By complying with the inclusion and exclusion criteria, literature screening, and data extraction were conducted.

**Results:**

A total of 19 randomized controlled articles involving 4501 cases were covered. As suggested from the meta-analysis, significant differences were reported with the 24-h urine protein (MD = −42.23, 95% confidence interval [CI] = -76.72 to −7.73, *P* = 0.02), urinary albumin-creatinine ratio (UACR) (MD = −23.57, 95% CI = −29.28 to −17.86, *P* < 0.00001), the systolic blood pressure (SBP) (MD = −2.73, 95% CI = −4.86 to −0.59, *P* = 0.01), and eGFR (MD = −1.56, 95% CI = −2.78 to −0.34, *P* = 0.01) in the subgroup of eplerenone vs placebo. The subgroups of eplerenone vs placebo (MD = 0.13, 95% CI = 0.07 to 0.18, *P* < 0.00001) and eplerenone vs thiazide diuretic (MD = 0.18, 95% CI = 0.13 to 0.23, *P* < 0.00001) showed the significantly increased potassium levels. However, no statistical significance was reported between the eplerenone treatment groups and the control in the effect exerted by serum creatinine (MD=0.03, 95% CI = −0.01 to 0.07, *P* = 0.12) and diastolic blood pressure (DBP) (MD = 0.11, 95% CI = −0.41 to 0.63, *P* = 0.68). Furthermore, significant risks of hyperkalemia were reported in the eplerenone group (K^+^ ≥ 5.5 mmol/l, RR = 1.70, 95%CI = 1.35 to 2.13, *P*=<0.00001; *K*+≥6.0 mmol/l, RR = 1.61, 95% CIs = 1.06 to 2.44, *P* = 0.02), respectively.

**Conclusions:**

Eplerenone has beneficial effects on CKD by reducing urinary protein and the systolic blood pressure, but it also elevates the risk of hyperkalemia.

## 1. Introduction

CKD has become a globally recognized serious public health problem and a major factor contributing to the global burden of disease [1]. At present, the common drugs for CKD treatment consist of ACEI or AREB, which are capable of reducing urine protein and protecting the kidneys by blocking the renin-angiotensin-aldosterone system. SGLT-2i and GLP-1 analogues offered unique renal protection in diabetic nephropathy as well. Besides, MRAs, acting on mineralocorticoid receptors and suppressing excessive MR activation, could be an effective supplementary treatment for the existing clinical treatment of CKD cases [2]. Though several nonsteroidal MRAs achieving fewer side effects were developed and studied, they have not yet achieved extensive clinical applications. Eplerenone, the second-generation selective MRAs, should be considered a treatment for CKD [3, 4]. Therefore, the clinical application value of CKD will be comprehensively analysed from the clinical indicators closely related to the prognosis of CKD.

Hence, we conducted this meta-analysis to systematically assess the second-generation selective MRAs eplerenone's safety and effectiveness for treating CKD.

## 2. Materials and Methods

### 2.1. Search Strategy

This meta-analysis protocol was performed in strict accordance with the preferred reporting items for systematic reviews and meta-analyses (PRISMA) statements [5]. A total of 4 databases (PubMed, The Cochrane Library, Embase, and Web of Science) were searched up to September 21, 2022. The search terms covered “chronic kidney disease,” “mineralocorticoid receptor antagonists,” and “eplerenone.”

### 2.2. Inclusion and Exclusion Criteria

#### 2.2.1. Inclusion Criteria

*(1) Participants*.

- Adult male and nonpregnant female cases diagnosed with CKD (GFR > 30 ml/min/1.73 m^2^, serum potassium (K^+^) ≤ 5.0 mmol/l in 24 h before randomization) [1].
- Study cases may or may not have had a history of mild to moderate hypertension (SBP/DBP ≥ 140/90 mmHg), whereas only SBP/DBP ≤ 180/110 mmHg and without symptomatic hypotension were eligible.
- All articles were randomized controlled trials (RCTs) and crossover articles, assessing the effect of eplerenone with or without ACEI/ARB, in comparison with placebo or active control as a treatment for CKD for at least 4 weeks.

*(2) Outcome measures*. The endpoints covered were 24-h proteinuria, urinary albumin-creatinine ratio (UACR), estimated glomerular filtration rate (eGFR), serum creatinine, systolic blood pressure (SBP), diastolic blood pressure (DBP), and serum potassium levels. Adverse effects (e.g., events of hyperkalemia, K^+^ ≥ 5.5 mmol/l and ≥6.0 mmol/l).

#### 2.2.2. Exclusion Criteria

(1) Duplicate published or incomplete literature; (2) meta-reviews, case reports, letters, meeting abstracts, etc.; (3) articles that cannot obtain the full text and the required outcome indicators; (4) nonhuman clinical trials and non-RCTs of eplerenone therapy; and (5) articles of CKD with an unclear diagnosis or combined with other diseases.

### 2.3. Study Selection and Data Extraction

By complying with the inclusion and exclusion criteria, two authors screened the title and abstract of the respective literature, and read the full text of the literature that met the criteria. Lastly, the data were extracted independently. Any disagreement between the 2 authors was addressed by consensus or by a third investigator. Data collection consisted of first authors, years and countries of literature, study designs, the No. of cases involved, interventions, study duration, as well as the endpoints of the study.

### 2.4. Evaluation of the Risk of Bias

Evaluation of the risk of bias was performed by the two authors with the use of the Cochrane Collaboration's risk of bias evaluation kit, covering the following items: (1) selective report (report bias); (2) incomplete outcome information (attribution bias); (3) blinding of outcome evaluation (detection bias); (4) blinding of participants and personnel (performance bias); (5) allocation concealment (selection bias); (6) random sequence generation (selection bias); and (7) other biases.

### 2.5. Statistical Analysis

Our study employed RevMan 5.3 software (the Cochrane Collaboration, UK) for this meta-analysis. With the use of the 95% confidence interval (CI) and mean difference (MD), the analysis was conducted on the continuous variable. Besides, the dichotomous variable had the expression of relative risk (RR) with a 95% CI. The *I*^2^ statistic was adopted for evaluating statistical heterogeneity, which was not considered to be significant when *I*^2^ < 50% and *P* > 0.1, and fixed‐effect model analyses were conducted for the data pooling; otherwise, the random‐effect model was chosen. A sensitivity investigation was carried out for evaluating the individual trial's contribution to the pooled effect through the sequential omission of the respective trial. The graphic data were presented through forest plots. And funnel plots were employed for testing publication biases.

## 3. Results

### 3.1. Search Results

**Figure 1:**
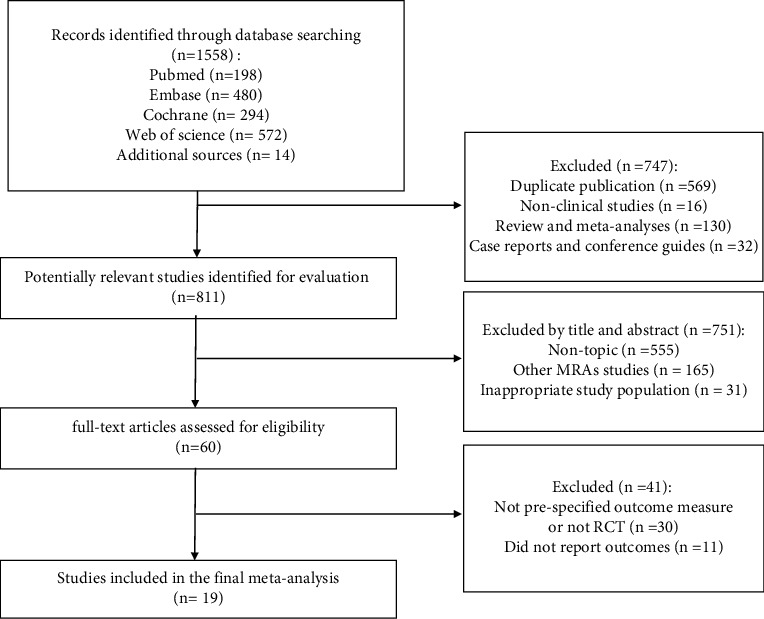
Flowchart of the literature search process.

After the retrieval of existing literature, 1558 eligible articles were obtained in total, and 19 articles [6–24] were covered here. The study selection is depicted in Figure 1.

### 3.2. Characteristics of Eligible Articles and Quality Evaluation

The final analysis involved 19 RCTs, published from 2002 to 2021, with a total of 4501 subjects. A total of 2296 cases were covered in the treatment group, and the control group involved 2205 cases. Among the 19 articles, the smallest one of the studies contained 15 cases, and the most involved 2127 cases. Intervention periods ranged from 8 weeks to 42 months. A summary of the basic characteristics of the literature is presented in Table 1.

**Figure 2:**
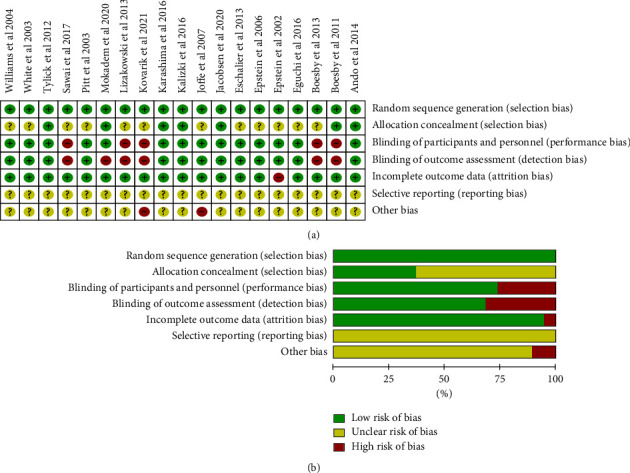
(a) Risk of bias summary: review authors judgements about the respective risk of bias item for each involved study. (b) Risk of bias graph: review researchers' judgements about the respective risk of bias item presented as percentages across all involved articles.

The Cochrane risk bias evaluation tool evaluates the quality exhibited by the involved literature, as illustrated in Figure 2. In the 19 articles involved, random sequence generation was all clear (100%), and 7 articles (36.8%) were assigned with adequate hiding. For the blinding design, 13 articles (68.5%) were suggested to be double-blind, 1 (5.2%) was single-blind, and 5 articles (26.3%) were nonblind. Among the 19 articles, 18 articles (94.7%) had clear results, one study lacked partial data, and except for 2 articles, which had other biases as impacted by the small sample size, the other articles were unclear about the bias of selective reporting and other biases.

### 3.3. Efficacy Outcomes

#### 3.3.1. Effect of Treatment on 24-h Proteinuria and UACR

The involved articles fell into subgroups by different control drugs (placebo/thiazide diuretic/RAS blockers/CCBs), the results of the meta-analysis included:

#### 3.3.2. The Effect of Treatment on 24-h Proteinuria

**Figure 3:**
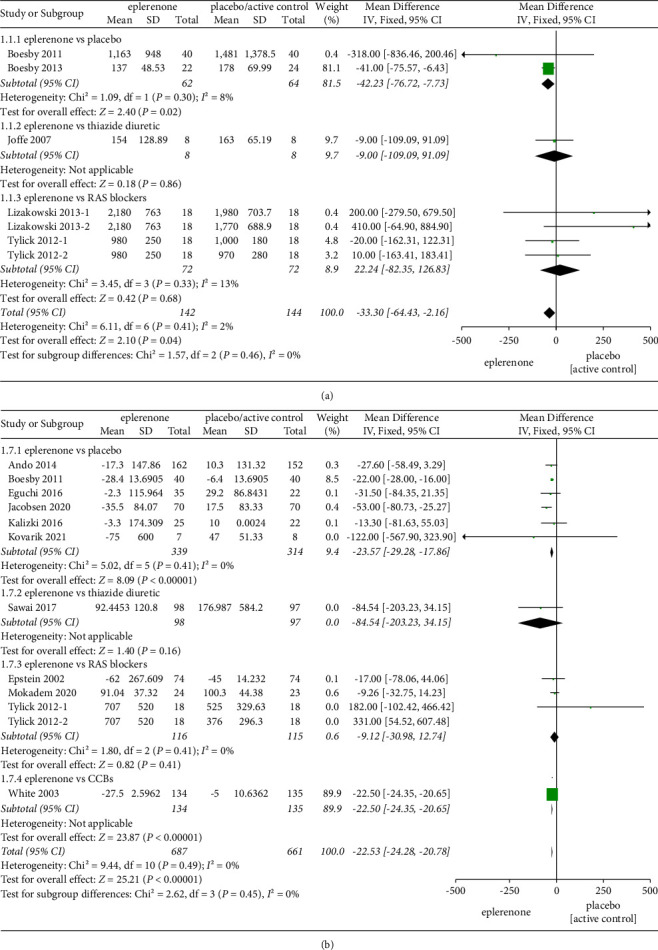
(a) Effect on 24-h proteinuria in articles comparing eplerenone to placebo/active control. (b) Effect on UACR for the eplerenone versus placebo/active control. The black diamond indicates summary information with the center of the estimate pooled with the median difference, and the width covers the relevant 95% CIs.

The effect of eplerenone on 24-h proteinuria was evaluated in 7 articles with 286 participants in total. No significant heterogeneity was identified between the articles involved in the subgroups (*χ*^2^ = 6.11, *P* = 0.41, *I*^2 ^= 2%), and the fixed effects model was adopted. A significant reduction was identified in the subgroup of eplerenone vs placebo (MD = −42.23, 95% CI = −76.72 to −7.73, *P* = 0.02), whereas no significant effect was identified compared with thiazide diuretic (MD = −9.0, 95% CI = −109.09 to 91.09, *P* = 0.86) and RAS blockers (MD = 22.24, 95% CI = −82.35 to 126.83, *P* = 0.68). Given all of the mentioned analysis, the total effect is that the 24-h proteinuria was significantly reduced in the eplerenone treatment groups than the control (MD = −33.30, 95% CI = −64.43 to −2.16, *P* = 0.04) (Figure 3(a)).

#### 3.3.3. The Effect of Treatment on UACR

Twelve articles showed the changes in UACR between the eplerenone treatment groups and the control. After the heterogeneity analysis, one study with high heterogeneity was excluded for further analysis, and the other 11 articles (*n* = 1348) did not indicate significant heterogeneity between the subgroups (*χ*^2^ = 9.44, *P* = 0.49, *I*^2^ = 0%). Significant reductions were identified in the subgroups of eplerenone vs placebo (MD = −23.57, 95% CI = −29.28 to −17.86, *P* < 0.00001) and eplerenone vs CCBs (MD = −22.50, 95% CI = −24.35 to −20.56, *P* < 0.00001), whereas no significant effect was identified in comparison with thiazide diuretic (MD = −84.54, 95% CI = −203.23 to 34.15, *P* = 0.16) and RAS blockers (MD = −9.12, 95% CI = −30.98 to 12.74, *P* = 0.41). Given all the mentioned analysis, the total effect was that the UACR was significantly reduced in the eplerenone treatment groups as compared with the control (MD = −22.53, 95% CI = −24.28 to −20.78, *P* < 0.00001) (Figure 3(b)).

#### 3.3.4. Effect of Treatment on eGFR

**Figure 4:**
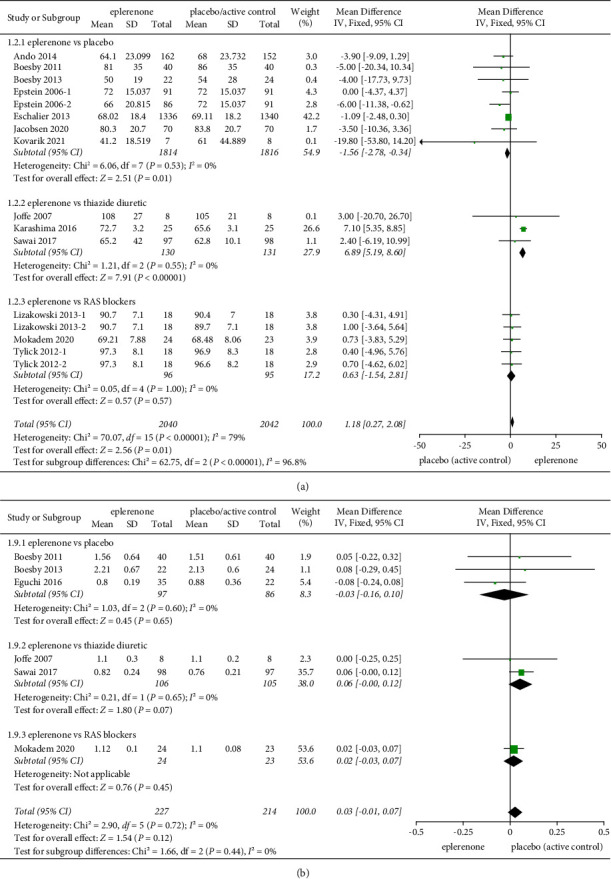
(a) Effect on eGFR in articles comparing eplerenone to placebo/active control. (b) Effect on serum creatinine for the eplerenone versus placebo/active control. The black diamond indicates summary information with the center of the estimate pooled with the median difference, and the width covers the relevant 95% CIs.

Sixteen articles (*n* = 4082) showed the changes in eGFR between the eplerenone treatment groups and the control. In the subgroup that compared eplerenone vs RAS blockers, no significant change was shown (MD = 0.63, 95% CI = −1.54 to 2.81, *P* = 0.57), while significant changes were observed in the subgroups of eplerenone vs placebo (MD = −1.56, 95% CI = −2.78 to −0.34, *P* = 0.01) and eplerenone vs thiazide diuretic (MD = 6.89, 95% CI = 5.19 to 8.60, *P* < 0.00001). In a pooled analysis of all 16 articles, there was high heterogeneity within the subgroups (*Chi*^*2*^ = 70.07, *P* < 0.00001, *I*^2^ = 79%), and the effect of eplerenone treatment on eGFR was significant compared with the control (MD = 1.18, 95% CI = 0.27 to 2.08, *P* = 0.01) (Figure 4(a)).

#### 3.3.5. Effect of Treatment on Serum Creatinine

The effect of eplerenone treatment on serum creatinine was evaluated in 6 articles (*n* = 441). No significant heterogeneity was identified between the articles involved in the subgroups (*Chi*^2^ = 2.90, *P* = 0.72, *I*^2^ = 0%). And no significant changes were observed in the subgroups, eplerenone vs placebo (MD=-0.03, 95% CI = −0.16 to 0.10, *P* = 0.65), eplerenone vs thiazide diuretic (MD=0.06, 95% CI = −0.00 to 0.12, *P* = 0.07), and eplerenone vs RAS blockers (MD = 0.02, 95% CI = −0.03 to 0.07, *P* = 0.45) (Figure 4(b)).

#### 3.3.6. Effect of Treatment on Blood Pressure

**Figure 5:**
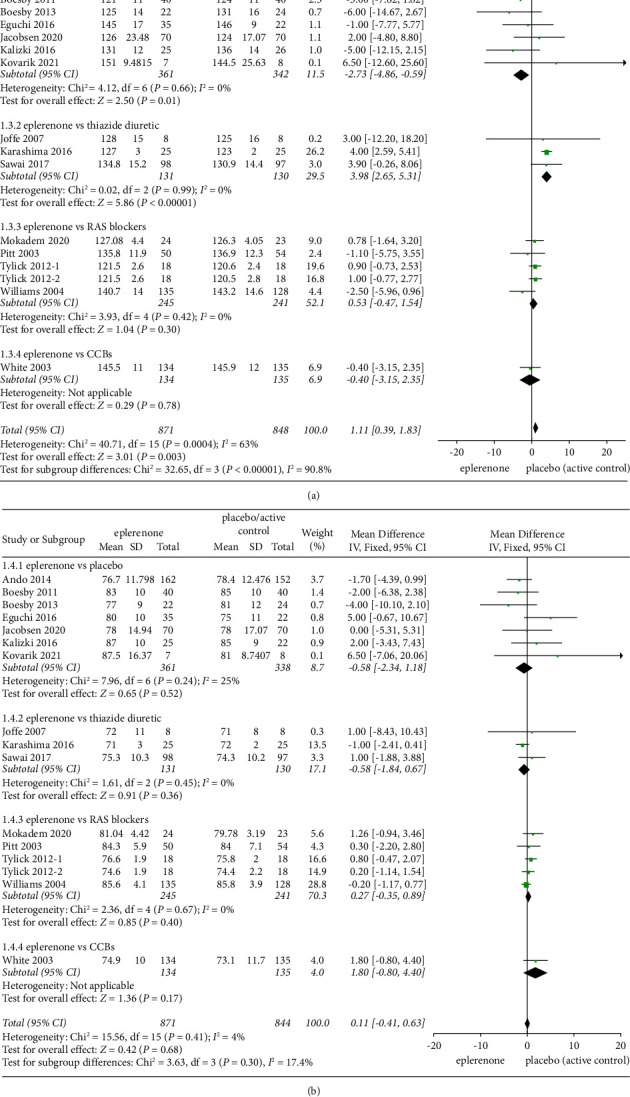
Forest plots of the effects on blood pressure between the eplerenone treatment groups and the control group. (a) Effect on SBP in articles comparing eplerenone to placebo/active control. (b) Effect on DBP in articles comparing eplerenone to placebo/active control. The black diamond indicates summary information with the center of the estimate pooled with the median difference, and the width covers the relevant 95% CIs.

The effect exerted by eplerenone treatment on blood pressure was evaluated in 16 articles (*n* = 1719) in total. In 7 articles comparing eplerenone to placebo, a significant change in SBP was reported in the eplerenone group (MD = −2.73, 95% CI = −4.86 to −0.59, *P* = 0.01), whereas in the subgroup of eplerenone vs thiazide diuretic, a significant change in SBP was reported in the control (MD = 3.98, 95% CI = 2.65–5.31, *P* < 0.00001), and no significant effect was identified compared with RAS blockers (MD = 0.53, 95% CI = −0.47 to 1.54, *P* = 0.30) and CCBs (MD = −0.40, 95% CI = −3.15 to 2.35, *P* = 0.78). In a pooled analysis of all articles, compared with placebo, the total effect slightly increased after eplerenone treatment (MD = 1.11, 95% CI = 0.39 to 1.83, *P* = 0.003). Furthermore, significant heterogeneity was found here among subgroups (*Chi*^2^ = 40.71, *P* = 0.0004, *I*^2^ = 63%) (Figure 5(a)).

However, in comparison with the control, the change of DBP (MD = 0.11, 95% CI = −0.41 to 0.63, *P* = 0.68) was not significantly different in the eplerenone treatment groups, and no heterogeneity was reported among subgroups (Chi^2^ = 15.56, *P* = 0.41, *I*^2^ = 4%). In articles comparing eplerenone vs placebo, DBP levels changed not significantly (MD = −0.58, 95% CI = −2.34 to 1.18, *P* = 0.52). Besides, the results in other articles were eplerenone vs thiazide diuretic (MD = −0.58, 95% CI = −1.84 to 0.67, *P* = 0.36), eplerenone vs RAS blockers (MD = 0.27, 95% CI = −0.35 to 0.89, *P* = 0.40), eplerenone vs CCBs (MD = 1.80, 95% CI = −0.80 to 4.40, *P* = 0.17) (Figure 5(b)), respectively.

#### 3.3.7. Effect of Treatment on Serum Potassium Levels

**Figure 6:**
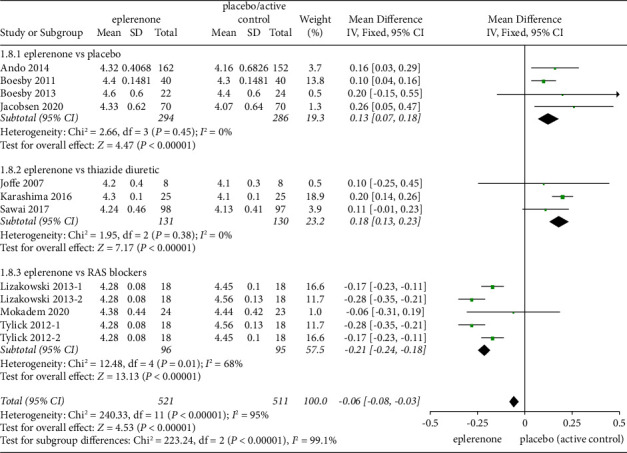
Forest plots of the effect on serum potassium levels in articles between the eplerenone treatment groups and the control group. The black diamond indicates summary information with the center of the estimate pooled with the median difference, and the width covers the relevant 95% CIs.

There were 12 articles (*n* = 1032) showing a difference in potassium levels between the eplerenone treatment groups and the control (MD=−0.06, 95% CI = −0.08 to −0.03, *P* < 0.00001). Significant rises in potassium levels were reported in the subgroups of eplerenone vs placebo (MD = 0.13, 95% CI = 0.07 to 0.18, *P* < 0.00001) and eplerenone vs thiazide diuretic (MD = 0.18, 95% CI = 0.13 to 0.23, *P* < 0.00001), whereas a lower potassium level was identified in the subgroup of eplerenone vs RAS blockers (MD = −0.21, 95% CI = −0.24 to −0.18, *P* < 0.00001) (Figure 6).

### 3.4. Adverse Events

#### 3.4.1. Hyperkalemia (≥5.5 mmol/l)

**Figure 7:**
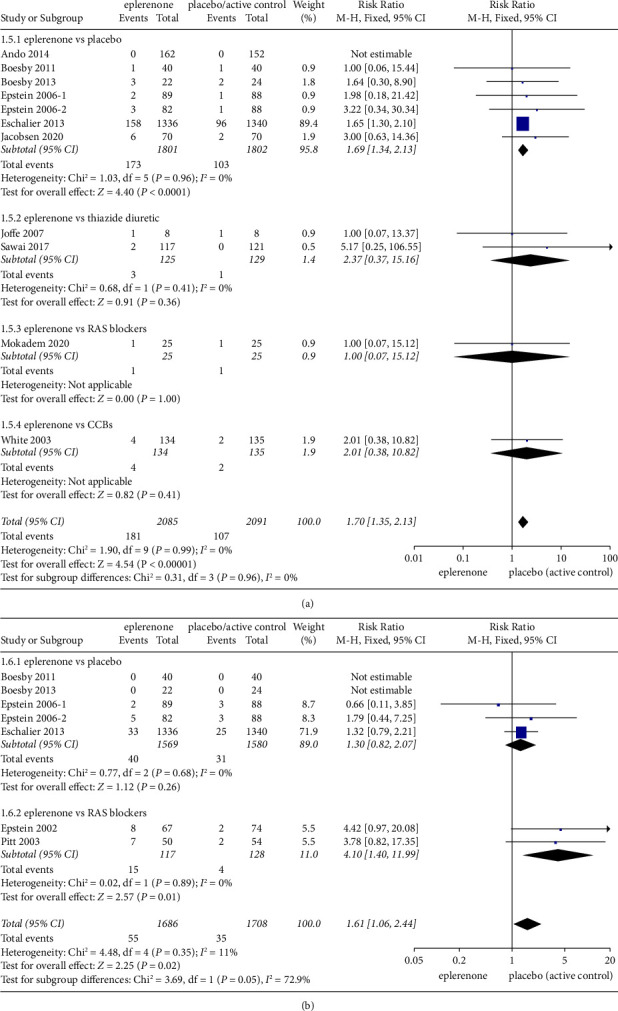
(a) Incidence of hyperkalemia (≥5.5 mmol/l) in articles comparing eplerenone to placebo/active control. (b) Incidence of hyperkalemia (≥6.0 mmol/l) in articles comparing eplerenone to placebo/active control. The black diamond represents summary data with the center of the estimate pooled with the risk ratio, and the width covers the corresponding 95% CIs.

A total of 10 articles involving 4176 patients suggested a difference between the eplerenone treatment groups and the control group regarding relative hyperkalemia risk (≥5.5 mmol/l). No significant difference was reported in the incidence of hyperkalemia in the subgroups of eplerenone vs thiazide diuretic (RR = 2.37, 95% CI = 0.37 to 15.16, *P* = 0.36), eplerenone vs RAS blockers (RR = 1.00, 95% CI = 0.07 to15.12, *P* = 1.00) and eplerenone vs CCBs (RR = 2.01, 95% CI = 0.38 to 10.82, *P* = 0.41), whereas in the subgroup of eplerenone vs placebo, a significant risk of hyperkalemia was reported in the eplerenone group (RR = 1.69, 95% CI = 1.34 to 2.13, *P* ≤ 0.00001). Moreover, in a pooled analysis of all articles, the total risk of hyperkalemia (≥5.5 mmol/l) in the eplerenone group increased by approximately 70% (RR = 1.70, 95% CI = 1.35 to 2.13, *P* ≤ 0.00001), and no significant heterogeneity was identified in the subgroup trials involved here (*Chi*^2^ = 1.90, *P* = 0.99, I^2^ = 0%) (Figure 7(a)).

#### 3.4.2. Hyperkalemia (≥6.0 mmol/l)

Seven articles (*n* = 3394) indicated the difference in risk of hyperkalemia (≥6.0 mmol/l) between the eplerenone treatment groups and the control group (RR = 1.61, 95% CI = 1.06 to 2.44, *P* = 0.02). No significant change was identified in the subgroup of eplerenone vs placebo (RR = 1.30, 95% CI = 0.82 to 2.07, *P* = 0.26), whereas in the subgroup of eplerenone vs RAS blockers, a significant risk of hyperkalemia was reported in the eplerenone group (RR = 4.10, 95% CI = 1.40 to 11.99, *P* = 0.01), and the total heterogeneity was low (Chi^2^ = 4.48, *P* = 0.35, *I*^2^ = 11%) (Figure 7(b)).

### 3.5. Evaluation of Sensitivity Analysis and Publication Bias

**Figure 8:**
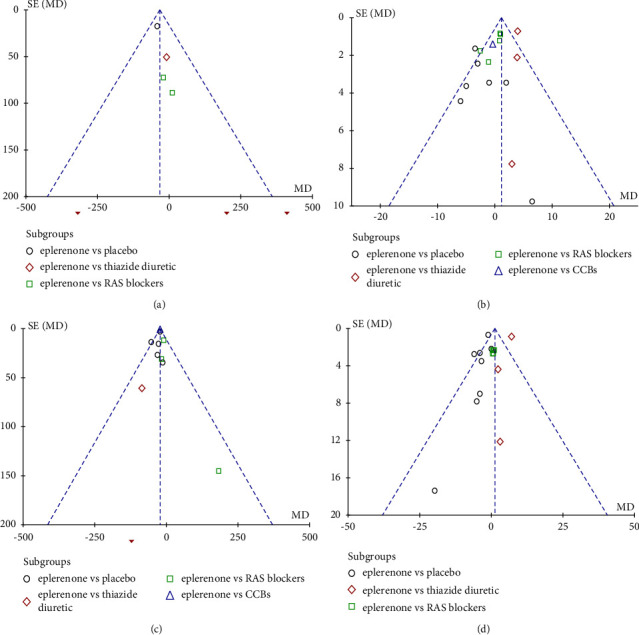
Funnel plots of the effects of eplerenone treatment on (a) 24-h proteinuria. (b) Systolic pressure. (c) UACR. (d) eGFR.

Sensitivity analysis of the involved articles showed no literature caused significant interference with the results of the meta-analysis, which meant the involved articles had good stability. The funnel plots were systematically performed with the effectiveness and adverse events indicators, including 24-h proteinuria, UACR, systolic pressure, and eGFR. As indicated in Figure 8, the distribution of the (A) to (D) funnel plots was symmetrical, and the scatter points of the study were mostly within the scope of the funnel plots, thereby demonstrating that the possibility of publication bias was insignificant.

## 4. Discussion

CKD results from diverse causes and is characterized by the progressive and irreversible loss of renal function, taking up about 11%–16% of the global population [25, 26]. Numerous basic and animal articles have verified that MRAs are capable of significantly suppressing the activity of proinflammatory cytokines and pro-oxidants, improving the anti-inflammatory response of kidney tissue, improving renal ischemia, mitigating collagen deposition, and preventing renal fibrosis [2, 27, 28]. Clinical articles also clearly indicate that MRAs are effective in treating chronic kidney disease. In this paper, we found that eplerenone has beneficial effects on CKD by reducing urinary protein and the systolic blood pressure, but with the risk of hyperkalemia at higher doses.

Articles have suggested that renal proteinuria predicts the acceleration of the course of progressive renal insufficiency and the shorter survival period of CKD cases [29]. Accordingly, reducing proteinuria can benefit the kidneys and reduce the risk of progression to ESRD [30, 31]. As suggested by the existing research summary. Eplerenone alone or in combination with ACEI/ARB can further significantly reduce proteinuria in cases suffering CKD and increase creatinine clearance, independent of its antihypertensive effects [28]. In this meta-analysis, compared with the placebo control, eplerenone treatment improved the 24-h urine protein and UACR levels of CKD cases, in line with the results of other existing articles [32]. However, this study revealed that no significant difference was reported in the efficacy of eplerenone treatment in comparison with the two controls of thiazide diuretic and RAS blockers in reducing urine protein. This finding is explained below. First, existing clinical trials and treatment experience have confirmed the major mechanism and beneficial effects of RAAS inhibitors to reduce urine protein for kidney protection. Second, no significant difference was reported between eplerenone and thiazide diuretics in reducing urine protein in this meta-analysis. Relevant literature articles suggested that thiazide drugs have a certain degree of urinary protein-lowering effect [33, 34], supporting the results of our study.

In this meta-analysis, the blood creatinine level of the eplerenone treatment group did not change significantly compared with the control group which is consistent with previous articles [35, 36]. However, articles have suggested that a small number of cases experienced deterioration in renal function after the addition of eplerenone treatment in CKD cases [37]. In comparison with the placebo group, the eGFR of the treatment group was reduced, and there were statistical differences, complying with the results of the above study. Considering that it is not correlated with the different baselines of the renal function of the subjects selected in the respective study, the renal function needs to be monitored in real time during the clinical application process. However, eGFR was more significantly reduced in the thiazide diuretic group, as compared with that of the eplerenone group. A prospective, multicentre clinical study (Cosmo-CKD) conducted in Japan demonstrated that, though hydrochlorothiazide can reduce urine protein and blood pressure [33], it reduces eGFR to a certain extent, complying with the results here. It is not considered to be correlated with increased aldosterone secondary to volume reduction [38].

In this meta-analysis, the effect of eplerenone on lowering SBP was better than that of the placebo group and weaker than that of the thiazide diuretic control and presented no significant difference in lowering blood pressure in comparison with the CCBs group. Additionally, no significant difference was reported between the changes in DBP and the controls. This result is inconsistent with the fact that eplerenone has a significant antihypertensive effect on SBD and DBP, as stated in some research results [32, 36]. The reasons for the difference between the results of this study and other articles are described as follows. (1) Thiazide diuretic suppresses the reabsorption of sodium chloride within the proximal and distal renal tubules, and it directly lowers blood pressure. Its antihypertensive effect may be stronger than the weaker diuretic effect of mineralocorticoid receptor antagonists, and this only targets the distal renal tubules and collecting ducts. (2) The comparison of the antihypertensive efficacy of eplerenone and CCBs is only involved in one study, with certain limitations. This can be clarified by further high-quality RCT articles. (3) In this meta-analysis, DBP was not significantly different from the control, and the changes were not excluded, which was correlated with the good baseline blood pressure control of the respective involved study. In addition, there is no significant heterogeneity in a single subgroup in this meta-analysis, while the high total heterogeneity is considered to be correlated with the difference in antihypertensive effect attributed to different drug choices in the control.

For adverse drug reactions, researchers (e.g., Sawai et al. [14], Ando et al. [15], Williams et al. [22], and Pitt et al. [23]) elucidated the drug treatment-related side effects during the treatment and the follow-up, thereby demonstrating the occurrence of no significant severe adverse reactions. El Mokadem et al. [11] also revealed that it is not common to discontinue medication as impacted by side effects in the respective treatment group. In this meta-analysis, only statistical analysis of changes in serum potassium levels was performed, and the risk of hyperkalemia during Eplerenone treatment was discussed. Compared with the control, eplerenone increased serum potassium levels, which was statistically significant from the clinical perspective. The risk of hyperkalemia fell to two levels of ≥5.5 mmol/l and ≥6.0 mmol/l for statistics. In the hyperkalemia risk (≥5.5 mmol/l) group, in comparison with the placebo, eplerenone treatment was accompanied by hyperkalemia risk. No significant difference was reported in other subgroups, complying with the results of some articles [32, 36, 37], demonstrating that MRAs may be associated with the risk of hyperkalemia. There are also articles verifying that there is no significant risk of high potassium in eplerenone treatment [39]. The reasons for the differences in the research results are analysed as follows. From one perspective, it is considered to be correlated with the increased risk of high potassium with the combined application of ACEI/ARB drugs in the involved articles. Moreover, the differences in the baseline blood potassium levels and baseline renal function of the populations involved in different articles are not excluded, and the risk of hyperkalemia is significantly increased in cases suffering high baseline blood potassium levels and poor renal function [40]. From another perspective, some articles have different definitions of the threshold of hyperkalemia risk (some are defined as ≥5.5 mmol/l, and some articles are defined as 6.0 mmol/l). However, eplerenone treatment is associated with the risk of hyperkalemia compared with the RAS blockers control. This is considered to be associated with the high-dose eplerenone treatment (100–200 mg) in the two selected articles in this treatment subgroup.

Lastly, this research has the following shortcomings. (1) For the quality exhibited by the involved articles, the sample size of some involved articles was low, and some research data was insufficient. (2) Only 13 articles (68.5%) were double-blind controlled articles, and only 36.8% of the articles mentioned allocation concealment. (3) In some articles, the observation period and follow-up period were short, thereby impacting the outcome indicators and making it unlikely for evaluating the effectiveness and safety in the long term. For this reason, more high-quality, large-sample randomized controlled clinical articles should be performed subsequently for further clarifying the effectiveness and safety of eplerenone for CKD cases.

## 5. Conclusion

In brief, this study suggested that eplerenone is capable of effectively lowering protein excretion in CKD cases and that it impacts blood pressure, with the blood creatinine kept stable during the treatment. But it can, to a certain extent, upregulate the blood potassium level and elevate the risk of hyperkalemia. So we need to screen the renal function and blood potassium level before treatment and formulate the drug treatment dose by complying with the principle of individualization. Thus, eplerenone can act as an effective supplement to the existing clinical treatment of cases suffering chronic kidney disease, but its long-term clinical efficacy and safety need to be further confirmed.

## Figures and Tables

**Table 1:** The characteristics of the involved observational articles.

No	Author	Year	Country	Study design	No. of patients included (Male/Female)	Interventions	Study duration	Endpoints
1	Epstein et al	2006	USA	Parallel RCT	T: 177 (116/61)	T: EPL + ACEI	12 weeks	BP, UACR, eGFR incidence of hyperkalemia
					C: 91 (50/41)	C: Placebo + ACEI		

2	Joffe et al	2007	USA	Cross‐over RCT	T: 8	T: EPL + ACEI	6 weeks	BP, 24 h-proteinuria, creatinine, estimated creatinine clearance, potassium, the incidence of hyperkalemia
					C: 8	C: HCTZ + ACEI		

3	Kovarik et al	2021	Austria	Parallel RCT	T: 7 (7/0)	T: EPL + ACEI	10 weeks	BP, UACR, eGFR
					C: 8 (5/3)	C: Placebo + ACEI		

4	Boesby et al	2013	Denmark	Parallel RCT	T: 22	T: EPL + ACEI/ARB	24 weeks	BP, 24 h-proteinuria, estimated creatinine clearance, eGFR, creatinine, potassium incidence of hyperkalemia
					C: 24	C: Placebo + ACEI/ARB		

5	Jacobsen et al	2020	Denmark	Parallel RCT	T: 70 (53/17)	T: EPL	26 weeks	BP, UACR, eGFR, potassium incidence of hyperkalemia
					C: 70 (53/17)	C: Placebo		

6	Mokadem et al	2020	Egypt	Parallel RCT	T: 25 (15/10)	T: EPL	24 weeks	BP, UACR, eGFR, creatinine, potassium incidence of hyperkalemia
					C: 25 (13/12)	C: ACEI		

7	Karashima et al	2016	Japan	Parallel RCT	T: 25 (17/8)	T: EPL + ARB	48 weeks	BP, UACR, eGFR, potassium
					C: 25 (17/8)	C: HCTZ + ARB		

8	Epstein et al	2002	USA	Parallel RCT	T: 74	T: EPL	24 weeks	BP, UACR, incidence of hyperkalemia
					C: 74	C: ACEI		

9	Sawai et al	2017	Japan	Parallel RCT	T: 98 (49/49)	T: EPL	8 weeks	BP, UACR, creatinine, eGFR, potassium incidence of hyperkalemia
					C: 97 (49/48)	C:Thiazide diuretic		

10	Ando et al	2014	Japan	Parallel RCT	T: 162 (114/48)	T: EPL + ACEI/ARB	52 weeks	BP, UACR, eGFR, potassium incidence of hyperkalemia
					C: 152 (100/52)	C: Placebo + ACEI/ARB		

11	Boesby et al	2011	Denmark	Cross‐over RCT	T: 40	T: EPL + ACEI/ARB	24 weeks	BP, 24 h-proteinuria, estimated creatinine clearance, eGFR, potassium incidence of hyperkalemia
					C: 40	C: Placebo + ACEI/ARB		

12	Eschalier et al	2013	France	Parallel RCT	T: 1055 (746/309)	T: EPL	42 months	eGFR, incidence of hyperkalemia
					C: 1072 (771/301)	C: Placebo		

13	Tylick et al	2012	Poland	Cross‐over RCT	T: 18	T: EPL + ARB	8 weeks	BP, UACR, eGFR, 24 h-proteinuria potassium
					C: 18	C1: Aliskiren + ARB		
						C2: ARB (double dose)		

14	Lizakowski et al	2013	Poland	Cross‐over RCT	T: 18	T: EPL + ARB	8 weeks	BP, eGFR, potassium
					C: 18	C1: Aliskiren + ARB		
						C2: ARB (double dose)		

15	Kalizki et al	2016	Germany	Parallel RCT	T: 25 (19/6)	T: EPL + ACEI/ARB	26 weeks	BP, UACR
					C: 26 (22/4)	C: Placebo + ACEI/ARB		
16	White et al	2003	China	Parallel RCT	T: 134 (61/73)	T: EPL	24 weeks	BP, UACR, incidence of hyperkalemia
					C: 135 (66/69)	C: Amlodipine		

17	Williams et al	2004	USA	Parallel RCT	T: 253 (150/103)	T: EPL	6 months	BP, incidence of hyperkalemia
					C: 246 (126/120)	C: ACEI		

18	Pitt et al	2003	USA	Parallel RCT	T: 50 (31/19)	T: EPL	9 months	BP, the incidence of hyperkalemia
					C: 54 (30/24)	C: ACEI		

19	Eguchi et al	2016	Japan	Parallel RCT	T: 35 (25/10)	T: EPL	12 weeks	BP, UACR, creatinine
					C: 22 (11/11)	C: Placebo		

## Data Availability

The clinical data of the population used to support the findings of this study are available from the corresponding author upon request.
